# Arthroscopic primary repair of proximal anterior cruciate ligament tears seems safe but higher level of evidence is needed: a systematic review and meta-analysis of recent literature

**DOI:** 10.1007/s00167-019-05697-8

**Published:** 2019-09-05

**Authors:** Jelle P. van der List, Harmen D. Vermeijden, Inger N. Sierevelt, Gregory S. DiFelice, Arthur van Noort, Gino M. M. J. Kerkhoffs

**Affiliations:** 1 Department of Orthopaedic Surgery, Spaarne Gasthuis Hospital, Hoofddorp, The Netherlands; 2 Amsterdam UMC, Department of Orthopaedic Surgery, University of Amsterdam, Amsterdam, The Netherlands; 3 Hospital for Special Surgery, Department of Orthopaedic Surgery, New York, USA; 4 Amsterdam UMC, Academic Center for Evidence Based Sports Medicine (ACES), University of Amsterdam, Amsterdam, The Netherlands; 5 Amsterdam UMC, Amsterdam Collaboration On Health and Safety in Sports (ACHSS), University of Amsterdam, IOC Research Center, Amsterdam, The Netherlands

## Abstract

**Purpose:**

To assess the outcomes of the various techniques of primary repair of proximal anterior cruciate ligament (ACL) tears in the recent literature using a systematic review with meta-analysis.

**Methods:**

PRISMA guidelines were followed. All studies reporting outcomes of arthroscopic primary repair of proximal ACL tears using primary repair, repair with static (suture) augmentation and dynamic augmentation between January 2014 and July 2019 in PubMed, Embase and Cochrane were identified and included. Primary outcomes were failure rates and reoperation rates, and secondary outcomes were patient-reported outcome scores.

**Results:**

A total of 13 studies and 1,101 patients (mean age 31 years, mean follow-up 2.1 years, 60% male) were included. Nearly all studies were retrospective studies without a control group and only one randomized study was identified. Grade of recommendation for primary repair was weak. There were 9 out of 74 failures following primary repair (10%), 6 out of 69 following repair with static augmentation (7%) and 106 out of 958 following dynamic augmentation (11%). Repair with dynamic augmentation had more reoperations (99; 10%), and more hardware removal (255; 29%) compared to the other procedures. All functional outcome scores were > 85% of maximum scores.

**Conclusions:**

This systematic review with meta-analysis found that the different techniques of primary repair are safe with failure rates of 7–11%, no complications and functional outcome scores of > 85% of maximum scores. There was a high risk of bias and follow-up was short with 2.1 years. Prospective studies comparing the outcomes to ACL reconstruction with sufficient follow-up are needed prior to widespread implementation.

**Level of evidence:**

IV.

## Introduction

Over the last year, there has been a renewed interest in the concept of primary repair of the anterior cruciate ligament (ACL) [76]. Open primary repair was commonly performed in the twentieth century and, despite promising short-term results [12, 21, 49, 50, 57, 68, 83], the outcomes were disappointing at longer follow-up [19, 22, 39, 40, 61, 69]. This resulted in an abandonment of the primary repair technique at that time and a shift towards ACL reconstruction that is still the gold standard for active and symptomatic patients today [51, 76].

There are multiple reasons why there has been renewed interest in primary repair following the disappointing historical results. First, historically all different tear types were treated with primary repair but several studies have suggested that primary repair should only be performed in selected patients with proximal tears, as there is better vascularity at the proximal end of the ligament [56, 74] and several studies shown better results of primary repair of proximal when compared to midsubstance tears [43, 72, 75, 76, 78, 81]. Another reason for the renewed interest is the lesser invasiveness of the surgery when compared to ACL reconstruction as no grafts are harvested or tunnels drilled, and thereby avoiding donor-site morbidity [6, 42] and earlier return to range of motion [77]. Finally, there have been several developments in surgical techniques, such as arthroscopic surgery, suture anchors, dynamic intraligamentary stabilization, and internal bracing, that were not available in the historical studies and this has also been a reason to reassess the outcomes of primary repair in the more recent era.

However, there are also objections to the renewed interest in primary repair. Given the disappointing historical outcomes of open primary repair, several surgeons have presumed that primary repair might be a risky procedure with higher failure rates than reconstruction [34, 63]. Furthermore, by performing primary repair in the more acute setting (for optimal tissue quality and prevention of ligament retraction), it is possible that too many ACL surgeries are performed, as some of the conservatively treated patients do well without ACL surgery [23, 53].

Recently, several small cohort studies have presented the first results of arthroscopic primary repair [1, 15, 16, 31, 33, 54]. This systematic review with meta-analysis was, therefore, performed to assess the safety and efficacy of the renewed primary repair techniques given the disappointing results in the historical literature. The goal of this study was to assess the outcomes of all techniques of primary repair in recent studies and abstracts and compare the outcomes between the different techniques. This study aims to provide an overview of the recent outcomes of various techniques of primary repair of proximal tears.

## Materials and methods

The Preferred Reporting Items for Systematic Reviews and Meta-Analyses (PRISMA) guidelines were followed when performing this study.

### Literature search

A systematic search was performed in the electronic search engines PubMed, Embase and Cochrane Library for studies reporting on outcomes of primary ACL repair. Following a preliminary search, the search algorithm “Anterior Cruciate Ligament AND (repair OR reinsertion OR reattachment OR healing OR suture)” was developed and used on July 2, 2019. The search was limited for studies reporting outcomes in the last 5 years (between January 1, 2014, and June 30, 2019) as recent systematic reviews have shown that no new studies have reported outcomes of modern primary repair before 2014 [72, 78, 81], and was limited to English studies.

After duplicate removal, two reviewers (JPL and HDV) first reviewed the title and abstract of all studies and then reviewed full texts of potential studies on the inclusion and exclusion criteria. References of full-text scanned studies were also reviewed for potentially interesting studies. Agreement was reached on the inclusion and exclusion of all studies and a third independent reviewer (AVN) was not required.

Inclusion criteria were (I) outcomes of primary repair with or without augmentation, (II) (mainly) treating proximal tears, (III) minimum 1-year follow-up and (IV) minimum level IV studies. Exclusion criteria were (I) long-term follow-up of historical studies [72, 78, 81], (II) not reporting tear location [7, 65], (III) treating multiligamentous knee injuries or knee dislocations, (IV) treating distal (bony) avulsion tears, (V) paediatric patient population [8, 24, 71], abstracts without full-text [3, 10, 13, 29] or (VI) multiple studies that report on the same group of patients (smallest cohort study or shortest follow-up excluded) [4, 15–18, 27, 28, 46, 47].

### Methodological quality of studies

Level of evidence of the included studies was assessed using the adjusted Oxford Centre for Evidence-Based Medicine 2011 Levels of Evidence [86]. The methodological quality of included studies was assessed using the Methodological Index for Non-Randomized Studies (MINORS) instrument [70], which is an instrument designed to assess methodological quality of both non-comparative and comparative studies. For this study, only the cohorts of primary repair were used and, therefore, only the non-comparative factors of the MINORS instrument were used. The strength of recommendation was determined using the Grades of Recommendation, Assessment, Development, and Evaluation (GRADE) Working Group system [5].

### Data extraction

All data were collected in Excel 2017 (Microsoft Corp., Redmond, WA, USA). Collected baseline characteristics data included author names, year of publication, number of patients at follow-up, length of follow-up, age, delay from injury to surgery and gender. Surgical techniques in the literature consisted of primary repair without augmentation, repair with static (suture (Internal Brace)) augmentation and repair with dynamic augmentation (Ligamys). For the repair without and with dynamic augmentation, the method of femoral fixation technique (transosseous tunnels or suture anchor) was also assessed. Collected outcomes consisted of failures (defined as rerupture or symptomatic instability), reoperations (defined as operation for other reason than revision), and removal of hardware (ROH; defined as removal of hardware without any other concomitant procedure). Furthermore, clinical stability consisting of Lachman and pivot shift test, and KT-1000 measurements (absolute measurements and percentage < 3 mm side-to-side difference) were collected. Collected outcome scores were International Knee Documentation Committee (IKDC) objective and subjective score [26], preinjury and postoperative Tegner score [73], Lysholm score [9], modified Cincinnati score [58, 66], Sports subscale of the Knee injury and Osteoarthritis Outcome Score (KOOS) [14], Single Assessment Numeric Score (SANE) on knee function [85], and visual analogue scale (VAS) for pain as these were most commonly reported and considered as relevant outcomes measures. Categorical outcomes were reported in percentages, and continuous outcomes were reported in mean ± standard deviation (SD). In case results were presented otherwise, transformation to means and SD was performed according to previously defined methods [32, 36, 82]. Pooled outcomes were collected for continuous outcomes by calculating weighted average and by calculating the incidence (e.g. total patients with KT-1000 side-to-side difference < 3 mm/total patients tested × 100%).

### Statistical analysis

Statistical analysis was performed using SPSS Statistics version 25.0 (SPSS Inc., Armonk, NY, USA) and Excel 2017. Differences in incidence were assessed using Pearson Chi-Square test and Fisher’s exact test (in case of expected values < 5). Continuous variables were not statistically compared, but the overall mean and standard deviations were calculated using standardized methods [32]. Forest plots were performed to assess differences for preinjury and postoperative Tegner activity levels by use of RevMan 5.3 and only studies reporting both preinjury and postoperative Tegner levels were included for this analysis. All tests were two sided and a *p *< 0.05 was considered statistically significant.

## Results

### Literature search

**Fig. 1:**
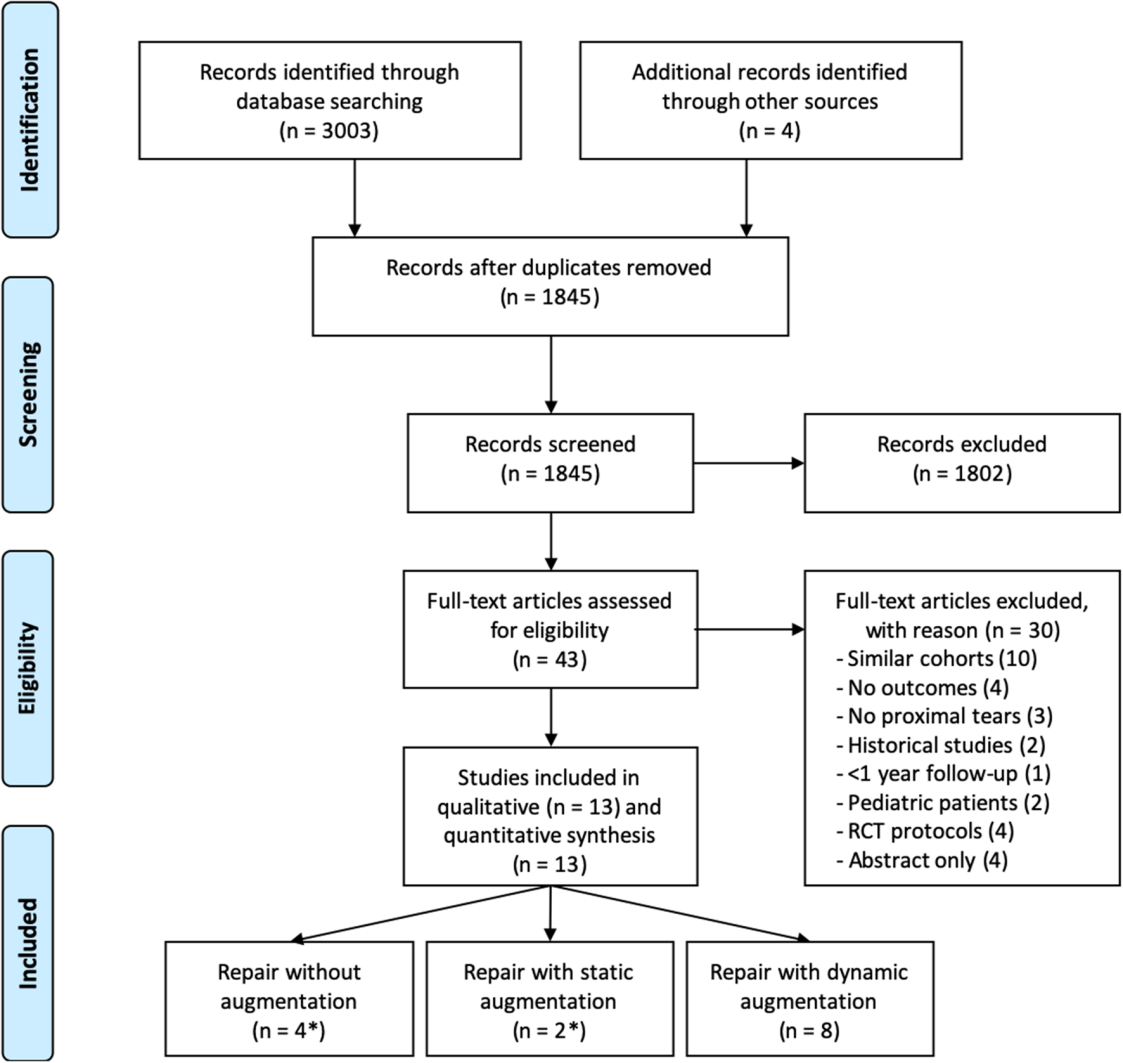
A PRISMA flowchart of the inclusion and exclusion of the study is shown. *One study reported outcomes of both primary repair with and without suture augmentation [38]

Eighteen hundred forty-five articles were screened on title and abstract for eligibility and 43 articles were reviewed on their full text for inclusion. A total of 13 studies reported on outcomes of primary repair and were included [1, 2, 11, 25, 31, 33, 35, 38, 41, 43, 52, 54, 62], of which, four used primary repair [1, 33, 38, 54], two used primary repair with suture augmentation [31, 38] (one reported outcomes of both with and without suture augmentation [38]) and eight used primary repair with dynamic augmentation (Fig. 1) [2, 11, 25, 35, 41, 43, 52, 62].

### Methodological quality of studies

One study was a level I study (8%) [35], there were no level II studies, two studies were level III studies (15%) [1, 38] and the majority (ten studies; 77%) were level IV studies [2, 11, 25, 31, 33, 41, 43, 52, 54, 62]. The recommendation for using primary repair for proximal ACL tears was weak using the GRADE system. The methodological quality of studies was graded according to the MINORS criteria (Table 1) and the average score was 10.9 out 16 points (68% of maximum). No blinding was applied in any of the studies and only two studies compared their results to ACL reconstruction [1, 35] of which one was a randomized controlled study (RCT) [35].

**Table 1:** Quality assessment of the included studies using the Methodological Index for Non-Randomized Studies (MINORS) criteria

Authors	Year	Journal/meeting	Evidence	Study design	1	2	3	4	5	6	7	8	Total
Achtnich et al. [1]	2016	Arthroscopy	III	Prospective	2	2	1	2	1	2	2	0	12
Ateschrang et al. [2]	2017	KSSTA	IV	Case series	2	2	2	2	0	1	1	0	10
Büchler et al. [11]	2016	Knee	IV	Case series	2	2	1	2	0	1	2	0	10
Häberli et al. [25]	2018	Knee	IV	Case series	2	2	1	2	0	2	2	0	11
Heusdens et al. [31]	2018	KSSTA	IV	Case series	2	2	2	1	0	1	2	1	11
Hoffmann et al. [33]	2017	J Orthop Surg Res	IV	Case series	2	2	0	2	1	2	2	0	11
Hoogeslag et al. [35]	2019	Am J Sports Med	I	RCT	2	2	2	2	1	2	2	2	15
Jonkergouw et al. [38]	2018	KSSTA	III	Retrospective	2	2	1	2	0	1	2	0	10
Kohl et al. [41]	2016	BJJ	IV	Case series	1	2	2	2	0	2	2	0	11
Krismer et al. [43]	2017	KSSTA	IV^a^	Case series	2	2	0	2	0	2	2	0	10
Meister et al. [52]	2017	KSSTA	IV	Case series	2	1	2	2	0	1	2	0	10
Mukhopadhyay et al. [54]	2018	Chin J Traumatol	IV	Case series	1	2	2	2	0	2	2	0	11
Osti et al. [62]	2019	KSSTA	IV	Case series	2	2	2	1	0	1	2	0	10

Only the non-comparative part of the MINORS criteria was used (i.e. first 8 questions). The criteria of MINORS [70] with 0 points when not reported, 1 when reported but not adequate, and 2 when reported and adequate. Maximum score is 16

1. A clearly stated aim: the question addressed should be precise and relevant in the light of available literature

2. Inclusion of consecutive patients: all patients potentially fit for inclusion (satisfying the criteria for inclusion) have been included in the study during the study period (no exclusion or details about the reasons for exclusion)

3. Prospective collection of data: data were collected according to a protocol established before the beginning of the study

4. End points appropriate to the aim of the study: unambiguous explanation of the criteria used to evaluate the main outcome which should be in accordance with the question addressed by the study. In addition, the end points should be assessed on an intention-to-treat basis

5. Unbiased assessment of the study end point: blind evaluation of objective end points and double-blind evaluation of subjective end points. Otherwise, the reasons for not blinding should be stated

6. Follow-up period appropriate to the aim of the study: the follow-up should be sufficiently long to allow the assessment of the main endpoint and possible adverse events

7. Loss to follow-up less than 5%: all patients should be included in the follow-up. Otherwise, the proportion lost to follow-up should not exceed the proportion experiencing the major end point

8. Prospective calculation of the study size: information of the size of detectable difference of interest with a calculation of 95% CI, according to the expected incidence of the outcome event, and information about the level for statistical

^a^This study reported being a level II study but we have classified this case series with failure analysis as level IV study

### Baseline characteristics

A total of 1101 patients in 13 different studies were included in this study with a mean age of 31 years, mean follow-up of 2.1 years, mean delay of 2 weeks and of which 60% were males.

Four studies performed arthroscopic primary repair without augmentation, of which in one study, two suture anchors were used to reattach the ACL back to the femoral footprint [38], in two studies, one suture anchor [1, 33], and in one study, transosseous tunnel fixation was used [54]. A total of 74 patients were included of which 63% were male. Mean age was 35 years, mean follow-up was 3.7 years and mean delay was 3 weeks. All patients had proximal tears (100%) (Table 2).

**Table 2:** Study characteristics with failure and reoperation rates of studies/abstracts reporting outcomes of arthroscopic primary ACL repair of proximal tears

Authors	Year	No. pts	FU (years)		Age (years)		Delay (wks)		Male (%)	Prox (%)	Fail. (%)	Reop. (%)	ROH (%)	Lachman		Pivot shift	
			Mn	Range	Mn	Range	Mn	Range						Neg (%)	Pos (%)	Neg (%)	Pos (%)
Primary repair without augmentation																	
Achtnich et al. [1]	2016	20	2.3	2.0–2.6	30		< 6^a^			100	15	5	0	85	15	80	20
Hoffmann et al. [33]	2017	12	6.6	5.0–8.2	43	19–67	1	0–3	25	100	25	0	0	75	25	75	25
Jonkergouw et al. [38]	2018	29	4.0	2.0–9.2	37	15–57	5	1–574	62	100	14	7	0				
Mukhopadhyay et al. [54]	2018	13	2.6	2.2–3.2	31	21–40	1	0–2	100	100	0	0	0	85	15	100	0
Primary repair with static augmentation																	
Heusdens et al. [31]	2018	42	2.0		33	14–60	< 13^a^		57	100	5	0	0				
Jonkergouw et al. [38]	2018	27	2.4	2.0–4.4	30	14–44	4	1–22	56	100	7	0	7				
Primary repair with dynamic augmentation																	
Ateschrang et al. [2]	2017	47	1.0		28		2		57	100	11	17					
Büchler et al. [11]	2016	45	1.0		26	18–54	2	0–3	72	73	7	0					
Häberli et al. [25]	2018	446	2.3	1.8–5.3	33		< 9^a^		56	73^b^	9	12	27				
Hoogeslag et al. [35]	2019	23	2.0		21	10–27	2	2–2	79	83	9	21	0	100	0		
Kohl et al. [41]	2016	50	2.0		30	18–50	2	0–3	68	80	10	18	60			90	10
Krismer et al. [43]	2017	264	> 2.0		31		2		59	77	14	2	35				
Meister et al. [52]	2017	26	1.0	1.0–1.2	28	18–50	2	1–4	65	62	15	20	8	73	27		
Osti et al. [62]	2019	57	1.0		28	15–54	2	0–4	65	84	18	23	18				
Total primary repair		**74**	**3.7**	**2.0–9.2**	**35**	**15–67**	**3**	**0–47**	**63**	**100**	**9**	**4**	**0**	**82**	**18**	**84**	**16**
Total repair with SA		**69**	**2.2**	**2.0–4.4**	**32**	**14–60**	**4**	**1–22**	**57**	**100**	**6**	**0**	**3**				
Total repair with DIS		**958**	**2.0**	**1.0–5.3**	**31**	**10–54**	**2**	**1–29**	**60**	**77**	**11**	**10**	**29**	**86**	**14**	**90**	**10**
Total		**1101**	**2.1**	**1.0–9.2**	**31**	**10–67**	**2**	**0–547**	**60**	**79**	**11**	**9**	**25**	**84**	**16**	**87**	**13**

No studies reported on the return to sport rate following primary repair at follow-up except

*No. pts* number of patients, *FU* follow-up in years, *wks* weeks, *Mn* mean, *prox.* percentage of patients with proximal tears, *reop.* reoperation, *ROH* removal of hardware, *RTS* return to sports, *Comp* complications, *Neg.* negative, *Pos* positive, *SA* suture augmentation

Bold values are the total values

^a^These studies only reported criteria such as operation within certain number of weeks

^b^Data collected from another study with same cohort of patients [28]

Two studies reported on outcomes of arthroscopic primary with static augmentation, of which in one study, transosseous tunnels for ACL fixation were used [31], and in one study, two suture anchors with suture augmentation in the proximal suture anchor were used [38] (Table 2). A total of 69 patients were included of which 57% were male. Mean age was 32 years, mean follow-up was 2.2 years and mean delay was 4 weeks (Table 2). All patients had proximal tears (100%).

Eight studies performed primary repair with dynamic augmentation on a total of 958 patients of which 60% were male. Mean age of these patients was 31 years, mean follow-up was 2.0 years and mean delay was 2 weeks. A total of 77% of patients had proximal tears (range 62–100%) (Table 2).

### Outcomes

**Fig. 2:**
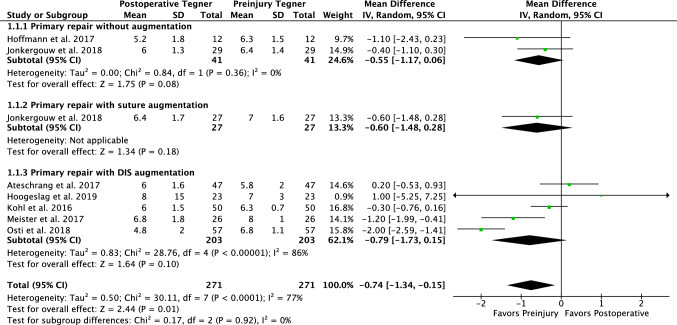
A Forest plot is shown with the preinjury and postoperative Tegner activity scores showing that a 0.7 level decrease in Tegner activity score can be expected following primary repair (regardless of technique; *p* = 0.01). The numbers on the right graph display the mean difference in Tegner score between preinjury and postoperative

In 74 patients who underwent primary repair without augmentation, the failure rate was 9%, additional reoperation rate 4%, and no ROH was reported. Eighty-two percent of patients had stable Lachman examination and 84% negative pivot shift (Table 2). Mean KT-1000 side-to-side difference was 1.9 ± 1.5 mm and 91% had < 3 mm side-to-side difference. Eighty-three percent had an IKDC objective score of A or B. The Tegner score changed from 6.4 ± 1.3 preinjury to 5.8 ± 1.4 postoperatively (Fig. 2), Lysholm score was 93 ± 11, modified Cincinnati was 91 ± 13, and the IKDC subjective was 90 ± 14 (Table 3).

**Table 3:** Functional and patient-reported outcomes of studies/abstracts reporting outcomes of arthroscopic primary ACL repair of proximal tears

Authors	Year	No. of pts	KT-1000		IKDC Objective					Tegner		Lysholm	Mod. Cinc	IKDC Subj	KOOS Sports	SANE	VAS pain
			Mn ± SD	< 3 mm (%)	A (%)	B (%)	C (%)	D (%)		Pre^b^	Post^b^						
Primary repair without suture augmentation																	
Achtnich et al. [1]	2016	20	2.0 ± 1.7		65	20	15	0									
Hoffmann et al. [33]	2017	12	2.1 ± 1.3	78	73	9	18	0		6.3 ± 1.5	5.2 ± 1.8	85 ± 20	84 ± 21	87 ± 17			
Jonkergouw et al. [38]	2018	29	1.9 ± 1.6^a^	88^a^	73	9	18			6.4 ± 1.4	6.0 ± 1.3	95 ± 8	94 ± 8	91 ± 12		89 ± 15	
Mukhopadhyay et al. [54]	2018	13	1.7 ± 0.7	100								95 ± 1					
Primary repair with static augmentation																	
Heusdens et al. [31]	2018	42													77 ± 31		1.3 ± 1.9
Jonkergouw et al. [38]	2018	27			72	17	11			7.0 ± 1.6	6.4 ± 1.7	93 ± 8	93 ± 10	89 ± 10		90 ± 10	
Primary repair with dynamic augmentation																	
Ateschrang et al. [2]	2017	47	2.1 ± 2.2		42	45	7		7	5.8 ± 2.0	6.0 ± 1.6	91 ± 8		86 ± 10			
Büchler et al. [11]	2016	45	0.0 ± 1.6	100							7.0 ± 1.1			90 ± 7			c
Häberli et al. [25]	2018	446								5.1 ± 1.5							
Hoogeslag et al. [35]	2019	23	1.2 ± 0.9	100	87	13	0		0	8.0 ± 1.5	7.0 ± 3.0			93 ± 14	78 ± 19		
Kohl et al. [41]	2016	50	1.2 ± 1.6							6.3 ± 0.7	6.0 ± 1.5	100 ± 1		99 ± 1			
Krismer et al. [43]	2017	264								6.8 ± 5.2							
Meister et al. [52]	2017	26		69	66	19	10		5	8.0 ± 1.0	6.8 ± 1.8	94 ± 11					
Osti et al. [62]	2019	57		51						6.8 ± 1.1	4.8 ± 2.0						
Total primary repair		**74**	**1.9 ± 1.5**	**91**	**83**		**17**			**6.4 ± 1.3**	**5.8 ± 1.4**	**93 ± 11**	**91 ± 13**	**90 ± 14**		**89 ± 15**	
Total repair with SA		**69**			**89**		**11**			**7.0 ± 1.6**	**6.4 ± 1.7**	**93 ± 8**	**93 ± 10**	**89 ± 10**	**77 ± 31**	**90 ± 10**	**1.3 ± 1.9**
Total repair with DIS		**958**	**1.0 ± 1.7**	**77**	**90**		**10**			**6.7 ± 1.5**	**6.1 ± 1.8**	**95 ± 6**		**92 ± 8**	**78 ± 19**		
Total		**1101**	**1.2 ± 1.6**	**77**	**87**		**13**			**6.7 ± 1.4**	**6.1 ± 1.7**	**94 ± 8**	**92 ± 12**	**91 ± 9**	**77 ± 27**	**90 ± 13**	**1.3 ± 1.9**

*No. of pts* number of patients, *IKDC* International Knee Documentation Committee score, *Mod. Cinc*., modified Cincinnati score, *KOOS* Knee injury and Osteoarthritis Outcome Score (sports subscale), *SANE* single assessment numeric evaluation, *VAS* visual analogue score, *Mn* mean, *SD* standard deviation

Bold values are the total values

^a^Data collected from another study with same cohort of patients [16]

^b^Only total sum calculated when both preinjury and postoperative Tegner score were reported

^c^Excluded due to inconsistency in data presentation

In 69 patients undergoing primary repair with static augmentation, the failure rate was 6%, additional reoperation rate 0% and ROH rate 3% (Table 2). Eighty-nine percent of patients had IKDC objective scores of A or B in one study. Tegner score changed from 7.0 ± 1.6 to 6.4 ± 1.7 in one study (Fig. 2). Lysholm score was 93 ± 8, modified Cincinnati 93 ± 10, IKDC subjective 89 ± 10, and KOOS Sports 77 ± 31 (Table 3).

In 958 patients undergoing primary repair with dynamic augmentation, the failure rate was 11%, additional reoperation rate 10%, and additional ROH 29%. Lachman examination was negative in 86% (two studies) and pivot shift was negative in 90% (one study) (Table 2). Mean KT-1000 examination was 1.0 ± 1.7 mm and 77% had < 3 mm side-to-side difference. Ninety percent had IKDC objective A or B. Tegner score changed from 6.7 ± 1.5 preinjury to 6.1 ± 1.8 postoperatively (Fig. 2), Lysholm score was 95 ± 6, and the IKDC subjective score was 92 ± 8 (Table 3).

### Differences between treatments

No differences were seen in failure rate between primary repair and repair with static augmentation (n.s.), between primary repair and dynamic augmentation (n.s.) nor between static and dynamic augmentation (n.s.). Primary repair with dynamic augmentation had more frequently reoperations when compared to primary repair with static augmentation (10% vs. 0%; *p* < 0.01), and had more frequently removal of hardware when compared to primary repair (29% vs. 0%; *p* < 0.01) and to repair with static augmentation (29% vs. 3%; *p* < 0.01). No differences between primary repair and repair with static augmentation were found for reoperations (n.s.) or ROH (n.s.). No clinically meaningful differences were noted in any of the functional and patient-reported outcome scores between all treatment groups. No studies reported on return to sports (RTS).

## Discussion

The main findings of this systematic review with meta-analysis were that the outcomes of primary repair have been reported in 1101 patients using three different techniques (primary repair, repair with static augmentation and repair with dynamic augmentation) and that the procedures seemed safe with failure rates of 7–11%, no complications and patient-reported outcomes of > 85% of the maximum scores. It was further noted that repair with dynamic augmentation leads to a higher reoperation rate (10%) and higher ROH rate (29%). Nearly all studies were retrospective case series with mean 2.1-year follow-up and there was a high risk of bias in these studies and, therefore, there was a low grade of recommendation for repair based on these studies.

Over the last few years, there has been a renewed interest in primary ACL repair and there are many reasons why the technique is being revisited. One of the main reasons for this renewed interest in primary repair is the strict patient selection that have been applied to the modern studies by only performing repairs on proximal tears [78, 81]. Historically, all tear types were repaired (of which most were midsubstance tears) and it is believed that this explains the disappointing historical outcomes of primary repair given the better vascularity and healing potential at the proximal and distal ends of the ligament [56, 74]. When reviewing the historical [78] and recent [20, 43] studies on primary repair, it has been shown that the outcomes of proximal tears are indeed better than repair of midsubstance tears. Another reason for the revisitation of primary repair is that historically surgery was performed using an arthrotomy, and the technique consisted of suturing the torn end of the ACL together [83] or using drill holes [22], whereas now suture anchors, static and dynamic augmentation and arthroscopic surgery are available.

When reviewing the failure rates in this study, it was noted that all three techniques reported acceptable failure rates ranging from 7 to 11% without statistical significant or clinically relevant differences between the techniques. It is difficult comparing these failure rates to the failure rates of ACL reconstruction in the literature as ACL reconstruction literature has more studies with higher level of evidence and larger number of patients. However, it seems that the failure rates of ACL reconstruction are generally lower than the failure rates of primary repair. In the Danish Registry, revision rates of ACL reconstruction at 2 years were 3%, although this registry only included revisions and not (non-operatively treated) failures [45], and failure rates in two large recent and meta-analyses were 7% for patients with an average age of 25 years [84, 87]. Two studies in this current study compared the outcomes of repair with reconstruction. Achtnich et al. compared 20 patients with ACL reconstruction to 20 patients with ACL repair for proximal tears, and noted similar outcomes in IKDC objective scores and KT-1000 stability with a higher failure rate in repair (15%) when compared to reconstruction (0%) [1]. Hoogeslag et al. recently performed a randomized controlled trial in which they compared the outcomes of 23 patients undergoing primary repair with dynamic augmentation with 21 patients undergoing ACL reconstruction [35]. They noted at follow-up similar patient-reported outcome scores and a higher failure rate of ACL reconstruction (19.0%) when compared to dynamic augmented repair (8.7%). When reviewing the overall pooled failure rates of primary repair in this study (7–11%), it seems that primary repair is a safe procedure with acceptable failure rates at short-term follow-up. It should be noted that these studies are mainly short-term follow-up, and more comparative studies with longer follow-up are necessary.

When reviewing reoperations in this study, reoperation rates of 0–10% were noted. A significantly higher reoperation rate was present following primary repair with dynamic augmentation compared to primary repair and repair with static augmentation. When reviewing dynamic augmentation, it is noted that most reoperations were due to scar tissue, range of motion deficits and arthrofibrosis. This might be explained by the additional spring device that is implanted in the tibia with this surgery. Similar to the reoperation rate, a higher removal of hardware rate was noted following dynamic augmentation compared to primary repair and primary repair with suture augmentation. When reviewing the study with the highest ROH rate by Kohl et al. [41] (60%), they stated that the tibial Ligamys implant was large and this led to the frequent removal of hardware in addition to the risk of arthrofibrosis [41]. The overall removal of hardware rate seems rather high with the dynamic augmentation procedure although it should be noted that not all patients had symptomatic ROH. Nonetheless, when combining failure rates, reoperation rates and ROH rates, more than half of dynamic augmentation repair patients had a complicated procedure, and future studies need to assess the additional value of the dynamic augmentation with these reoperation rates, especially given the findings in this study that the failure rates or reoperations rates were not lower with dynamic augmentation repair.

Interestingly, none of the studies reported return to sport (RTS) rates following any of the techniques besides the Tegner activity scale. It is possible that this has not been reported due to the small sample size of the studies and the relatively new surgical technique. The Tegner activity level dropped on average from 6.6 pre-injury to 5.9 at follow-up but future studies assessing the RTS as this is one of the main goals of ACL surgery [44].

Besides the aforementioned potential advantages of primary repair, there are also potential disadvantages of primary repair. Since primary repair needs to be performed in the (sub)acute setting to prevent ligament retracting and to optimize tissue quality [55, 59, 60], patients will be operated without attempting conservative treatment first and this will likely result in performing ACL surgery in a subset of patients that do not need ACL surgery. Some guidelines recommend attempting conservative treatment first in patients that do not return to pivoting sports or are willing to adjust their activity level as some of the conservatively treated patients can cope and do not need ACL surgery [23, 53]. On the contrary, treating patients conservatively or delaying the interval between injury and surgery increases the chance of meniscus and chondral damage [30, 37, 53, 64] and several studies have shown that meniscus damage and meniscectomy increases the rate of osteoarthritis at longer follow-up [48, 67]. A study by Sanders et al. showed at 14-year follow-up that performing ACL reconstruction decreases the risk of secondary meniscus tears, subsequent osteoarthritis and the need for total knee arthroplasty when compared to treating ACL injuries conservatively [64]. Ideally, it should be identified early which patients require surgery to decrease the chance of secondary meniscus or chondral damage, improve outcomes of ACL reconstruction [30], and ultimately decrease the risk of osteoarthritis at longer-term follow-up [48, 64, 67]. Potentially, in these patients, there might also be a role for primary repair in case a proximal tear is found during surgery, which is estimated to occur in approximately 15–40% of patients with acute ACL tears [79, 80].

Limitations of this study are present. First of all, most included studies in this review were of retrospective nature and had no control group and, therefore, no direct comparison between different treatments could be performed. This made it impossible to avoid or decrease potential bias, such as selection bias of which patients were treated with repair and publication bias. It should be mentioned, however, that there were two well-performed studies that compared their outcomes with ACL reconstruction and more of these studies are needed [1, 35]. Second, not all patients in the dynamic augmentation group had proximal tears which could influence the outcomes of dynamic augmentation repair. When considering that better outcomes of dynamic augmentation repair have been reported in patients with proximal tears [43], it should be noted better outcomes are expected when only patients with proximal tears are treated in the dynamic augmentation studies. Furthermore, the total number of patients in this study were small due to the relatively “new” treatment, which prevents drawing hard conclusions on the pooled outcomes. Finally, no correction for potential confounders such as concomitant injuries (e.g. meniscus or chondral injuries), age, gender, level of activity, or length of follow-up could be performed due to the relatively low number of patients and these could significantly influence outcomes. Despite these limitations, this study is the first to provide an overview of the recent outcomes of various techniques of primary repair of proximal tears and the current level of evidence that is available on primary repair.

## Conclusion

This systematic review with meta-analysis found that the different techniques of primary repair (primary repair without augmentation, with static and with dynamic augmentation) were safe with failure rates between 7 and 11%, and good functional outcome scores in 1101 patients. Higher reoperation rates (10%) and removal of hardware rates (29%) were noted with dynamic augmentation repair. Nearly all studies were retrospective without a control group and possessed a high risk of bias and prospective studies comparative studies with sufficient follow-up are needed prior to widespread implementation.
