# Correction to: Arthroscopic primary repair of proximal anterior cruciate ligament tears seems safe but higher level of evidence is needed: a systematic review and meta-analysis of recent literature

**DOI:** 10.1007/s00167-019-05757-z

**Published:** 2019-11-06

**Authors:** Jelle P. van der List, Harmen D. Vermeijden, Inger N. Sierevelt, Gregory S. DiFelice, Arthur van Noort, Gino M. M. J. Kerkhoffs

**Affiliations:** 1 Department of Orthopaedic Surgery, Spaarne Gasthuis Hospital, Hoofddorp, The Netherlands; 2 Amsterdam UMC, Department of Orthopaedic Surgery, University of Amsterdam, Amsterdam, The Netherlands; 3 Hospital for Special Surgery, Department of Orthopaedic Surgery, New York, USA; 4 Amsterdam UMC, Academic Center for Evidence Based Sports Medicine (ACES), University of Amsterdam, Amsterdam, The Netherlands; 5 Amsterdam UMC, Amsterdam Collaboration On Health and Safety in Sports (ACHSS), University of Amsterdam, IOC Research Center, Amsterdam, The Netherlands

## Correction to: Knee Surgery, Sports Traumatology, Arthroscopy 10.1007/s00167-019-05697-8

Unfortunately, the original publication of this article contains an error in table 1. The revised version of Table 1 is updated here. The original article has been corrected.

**Table 1:** Quality assessment of the included studies using the Methodological Index for Non-Randomized Studies (MINORS) criteria

Authors	Year	Journal/meeting	Evidence	Study design	1	2	3	4	5	6	7	8	Total
Achtnich et al. [1]	2016	Arthroscopy	III	Prospective	2	2	1	2	1	2	2	0	12
Ateschrang et al. [2]	2017	KSSTA	IV	Case series	2	2	2	2	0	1	1	0	10
Büchler et al. [11]	2016	Knee	IV	Case series	2	2	1	2	0	1	2	0	10
Häberli et al. [25]	2018	Knee	IV	Case series	2	2	1	2	0	2	2	0	11
Heusdens et al. [31]	2018	KSSTA	IV	Case series	2	2	2	1	0	1	2	1	11
Hoffmann et al. [33]	2017	J Orthop Surg Res	IV	Case series	2	2	0	2	1	2	2	0	11
Hoogeslag et al. [35]	2019	Am J Sports Med	I	RCT	2	2	2	2	1	2	2	2	15
Jonkergouw et al. [38]	2018	KSSTA	III	Retrospective	2	2	1	2	0	1	2	0	10
Kohl et al. [41]	2016	BJJ	IV	Case series	1	2	2	2	0	2	2	0	11
Krismer et al. [43]	2017	KSSTA	IV^a^	Case series	2	2	0	2	0	2	2	0	10
Meister et al. [52]	2017	KSSTA	IV	Case series	2	1	2	2	0	1	2	0	10
Mukhopadhyay et al. [54]	2018	Chin J Traumatol	IV	Case series	1	2	2	2	0	2	2	0	11
Osti et al. [62]	2019	KSSTA	IV	Case series	2	2	2	1	0	1	2	0	10

Only the non-comparative part of the MINORS criteria was used (i.e. first 8 questions). The criteria of MINORS [70] with 0 points when not reported, 1 when reported but not adequate, and 2 when reported and adequate. Maximum score is 16

1. A clearly stated aim: the question addressed should be precise and relevant in the light of available literature

2. Inclusion of consecutive patients: all patients potentially fit for inclusion (satisfying the criteria for inclusion) have been included in the study during the study period (no exclusion or details about the reasons for exclusion)

3. Prospective collection of data: data were collected according to a protocol established before the beginning of the study

4. End points appropriate to the aim of the study: unambiguous explanation of the criteria used to evaluate the main outcome which should be in accordance with the question addressed by the study. In addition, the end points should be assessed on an intention-to-treat basis

5. Unbiased assessment of the study end point: blind evaluation of objective end points and double-blind evaluation of subjective end points. Otherwise, the reasons for not blinding should be stated

6. Follow-up period appropriate to the aim of the study: the follow-up should be sufficiently long to allow the assessment of the main endpoint and possible adverse events

7. Loss to follow-up less than 5%: all patients should be included in the follow-up. Otherwise, the proportion lost to follow-up should not exceed the proportion experiencing the major end point

8. Prospective calculation of the study size: information of the size of detectable difference of interest with a calculation of 95% CI, according to the expected incidence of the outcome event, and information about the level for statistical

^a^This study reported being a level II study but we have classified this case series with failure analysis as level IV study

